# Supplementary material for: Directional reorientation of migrating neutrophils is limited by suppression of receptor input signaling at the cell rear through myosin II activity
Source: Nat Commun. 2021 Nov 16;12:6619. doi: 10.1038/s41467-021-26622-z (PMC8595366; doi:10.1038/s41467-021-26622-z)
Supplement: Supplementary file 1 — Supplementary Information [file 41467_2021_26622_MOESM1_ESM.pdf]

Supplementary Information

Directional reorientation of migrating neutrophils is limited by suppression of receptor input signaling at the cell rear through myosin II activity

Amalia Hadjitheodorou[1,2], George R. R. Bell[3], Felix Ellett[4], Shashank Shastry[3], Daniel Irimia[4], Sean R. Collins[3,*], and Julie A. Theriot[2,*]

[1]Department of Bioengineering, Stanford University, Stanford, CA, USA

[2]Department of Biology and Howard Hughes Medical Institute, University of Washington, Seattle, WA, USA

[3]Department of Microbiology and Molecular Genetics, University of California, Davis, Davis, CA, USA

[4]Department of Surgery, BioMEMS Resource Center, Massachusetts General Hospital, Harvard Medical School, Boston, MA, USA

*For correspondence: jtheriot@uw.edu, srcollins@ucdavis.edu

Supplementary Figure 1

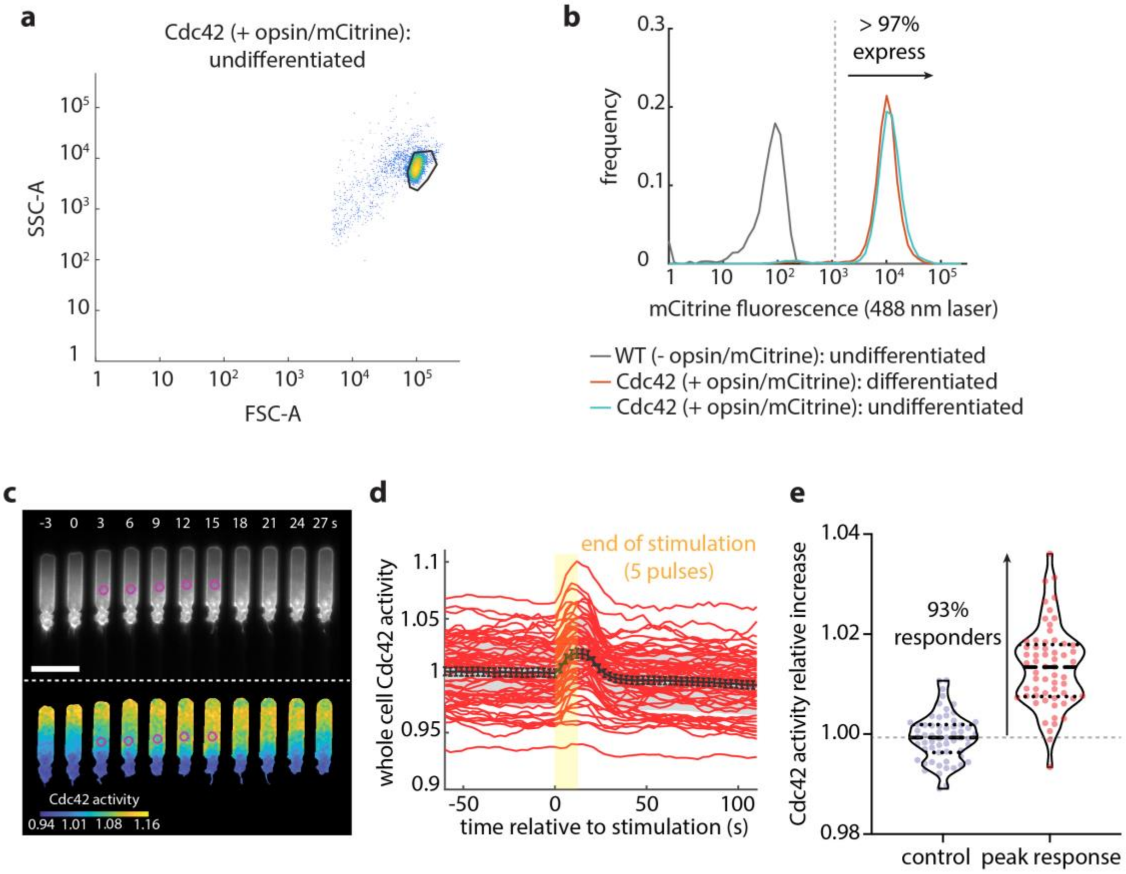

Supplementary Figure 1: Flow cytometry and center stimulation assay reveal that almost all cells are expressing the opsin receptor and that activated receptors signal to Cdc42.

(a) Example of gating strategy for flow cytometry measurements. **(b)** Flow cytometry measurement for mCitrine fluorescence of *n*=9972 differentiated (red) and *n*=10019 undifferentiated (cyan) HL60 cells expressing parapinopsin (tagged with mCitrine) and a red/far-red Cdc42 FRET sensor. Wild type cells (*n*=9904) not expressing the opsin used as control (grey). **(c)** Live-cell imaging snapshots of a representative center stimulation experiment on an HL60 cell expressing parapinopsin and the Cdc42 FRET sensor. Cells migrate unperturbed for 60 s before administering 5 pulses at their centroid (magenta circles). Upper and lower panels show the registered sensors (grey scale) and the computed Cdc42 activity, respectively. Images were captured every 3 s and subsampled for illustration purposes. Scale bar: 25 µm. **(d)** Mean Cdc42 activity averaged over the entire cell body over time (*n*=60 cells from 6 independent experiments) stimulated 5 times at their centroid (red lines: individual cells, black line: mean, grey shaded region: SD, error bars: mean value ± SE). Rectangular yellow shaded region represents the start and end of the 5-pulse stimulation. **(e)** Violin plots of the relative increase of Cdc42 activity over a control zone and over the peak response zone for the same cells shown in **(d)**. Dashed grey line represents the threshold as defined by the median of the control response distribution. Source data are provided as a Source Data file.

Supplementary Figure 2

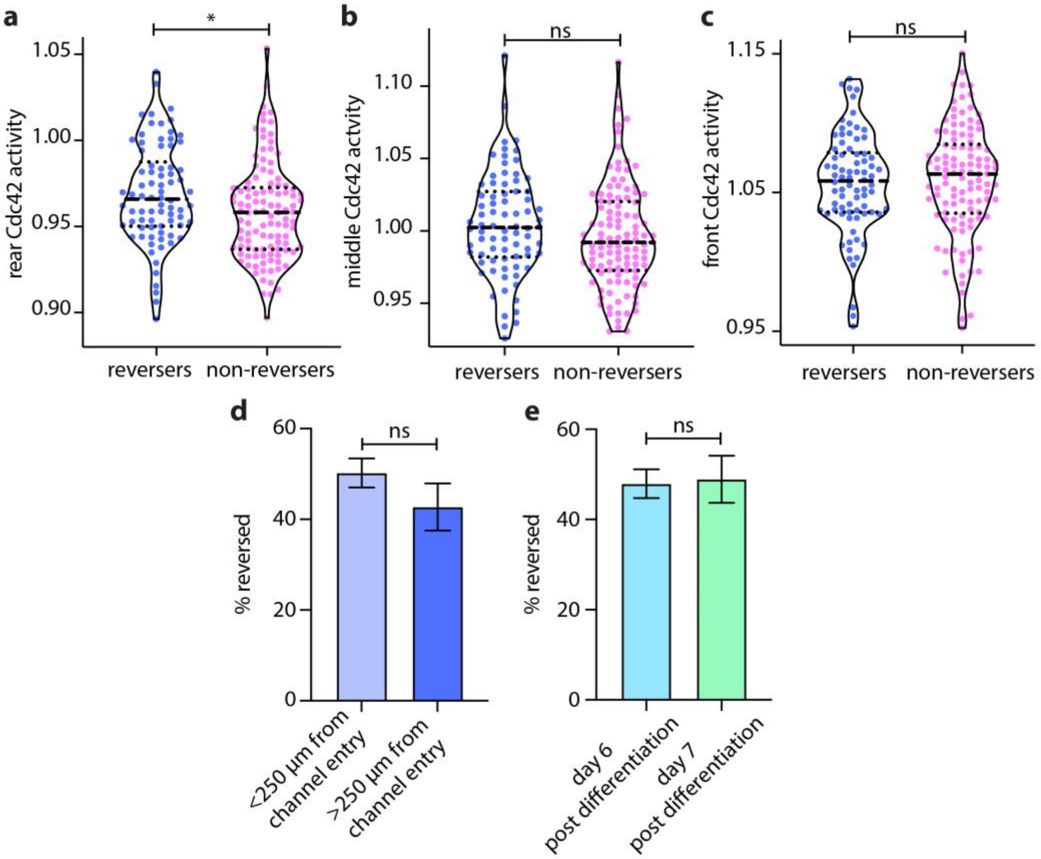

Supplementary Figure 2: Subcellular analysis reveals that non-reversers have a stronger rear as compared to reversing cells.

(a-c) Violin plots of mean cell rear **(a)**, cell middle **(b)**, and cell front **(c)** Cdc42 activity of *n*=78 reversers and *n*=110 non-reversers, averaging over 15 s prior to initiating persistent rear stimulation; *p*-values of two-sided Wilcoxon rank sum test (* represents $p<0.05$, ns represents $p>0.05$). **(d-e)** Bar plots of the percentage of cells that reversed stratified by the distance from the channel entrance **(d)**; closer to the channel entry: distance from entry <250 µm (*n*=247 cells), farther from channel entry: distance>250 µm (*n*=89 cells) as well as stratified by their differentiation age **(e)**: day 6 (*n*=244 cells), day 7 (*n*=92 cells). Error bars represent confidence intervals assuming a binomial distribution around the cumulative mean of each group and two-sided Fisher exact test revealed no significant difference between the compared conditions ($p>0.05$). Source data are provided as a Source Data file, including exact *p*-values.

Supplementary Figure 3

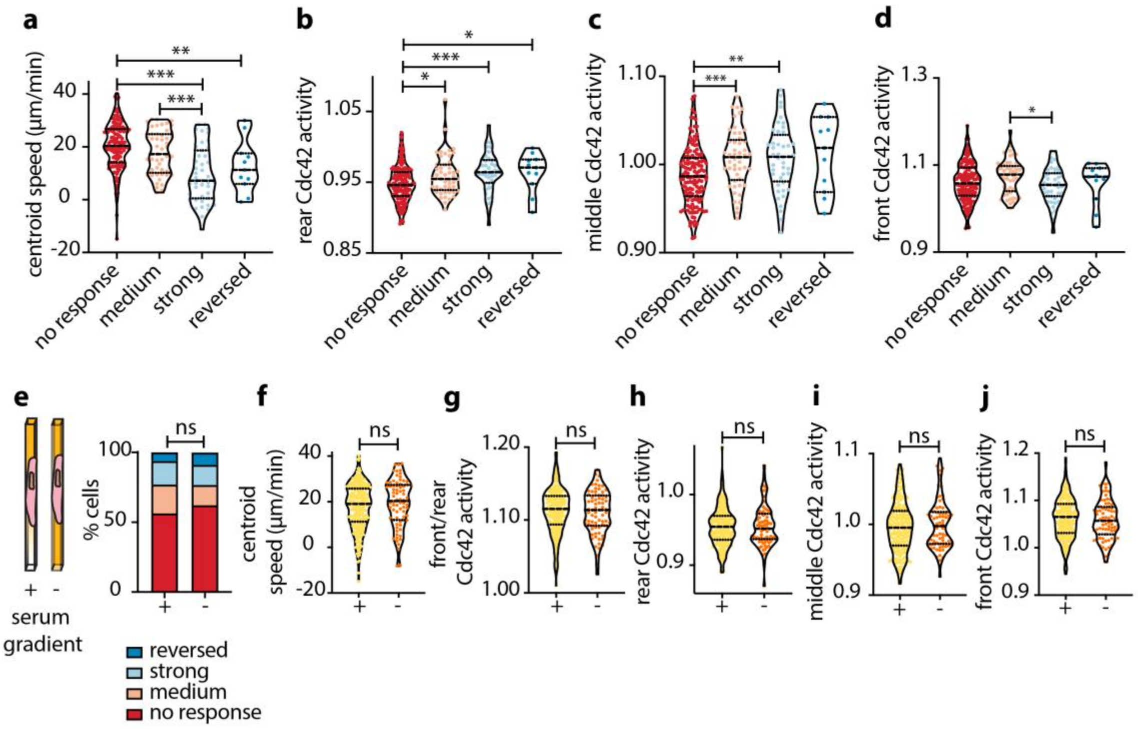

Supplementary Figure 3: Behavioral responses are due in part to pre-existing variation in Cdc42 polarity, which is independent of the serum gradient.

(a-d) Violin plots of mean centroid speed **(a)**, and mean cell rear **(b)**, cell middle **(c)**, and cell front **(d)** Cdc42 activity of n=141 non-responders, n=49 medium responders, n=44 strong responders, and n=11 reversers, averaging over 15 s prior to initiating 12-pulse stimulation; p-values of two-sided Wilcoxon rank sum test (*: $p<0.05$, **: $p<0.01$, ***: $p<0.001$, pairs not shown have $p>0.05$). **(e)** Stacked bar plots of the percentage of cells that showed no response, medium response, strong response and reversed for n=261 cells that migrated up a serum gradient and for n=101 cells that migrated in a homogeneous serum environment (from 20 and 6 independent experiments, respectively), two-sided Fisher exact test revealed no significant difference between the two conditions ($p>0.05$). **(f-j)** Violin plots of mean centroid speed **(f)**, and mean front/rear **(g)**, cell rear **(h)**, cell middle **(i)**, and cell front **(j)** Cdc42 activity of n=245 cells that migrated up a serum gradient and n=75 cells that migrated in a homogeneous serum environment, averaging over 15 s prior to initiating 12-pulse stimulation; ns represents $p>0.05$ of two-sided Wilcoxon rank sum test. Source data are provided as a Source Data file, including exact p-values.

Supplementary Figure 4

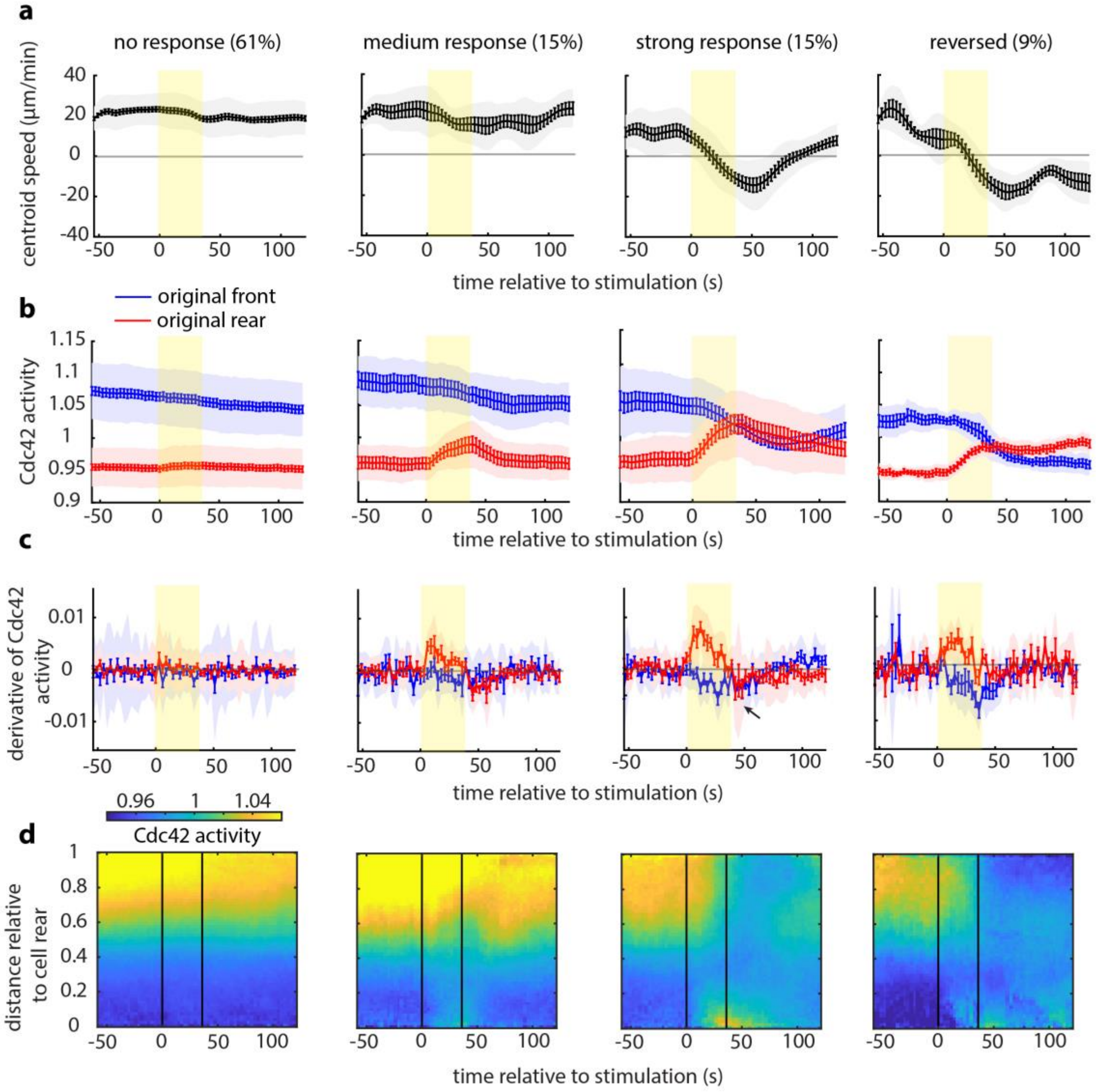

Supplementary Figure 4: Migratory and signaling responses are qualitatively similar with and without a serum gradient.

(a-c) Cell centroid speed **(a)**, mean Cdc42 activity **(b)** and derivative of Cdc42 activity **(c)** at the original front (blue) and rear (red) over time for each cellular response (lines: means, shaded regions: SD, error bars: mean value ± SE). Data are averages from *n*=46 non-responders, *n*=9 medium responders, *n*=13 strong responders, and *n*=7 reversers that migrated in a homogeneous serum environment from 6 independent experiments. Rectangular yellow shaded region marks the start and end of the 12-pulse stimulation. **(d)** Average kymograph representation of Cdc42 activity as a function of time (*x*-axis) and vertical position relative to the cell rear (*y*-axis) for non-responding, medium responding, strong responding and reversing cells. Vertical black lines indicate the start and end of the pulsated stimulation. Source data are provided as a Source Data file.

Supplementary Figure 5

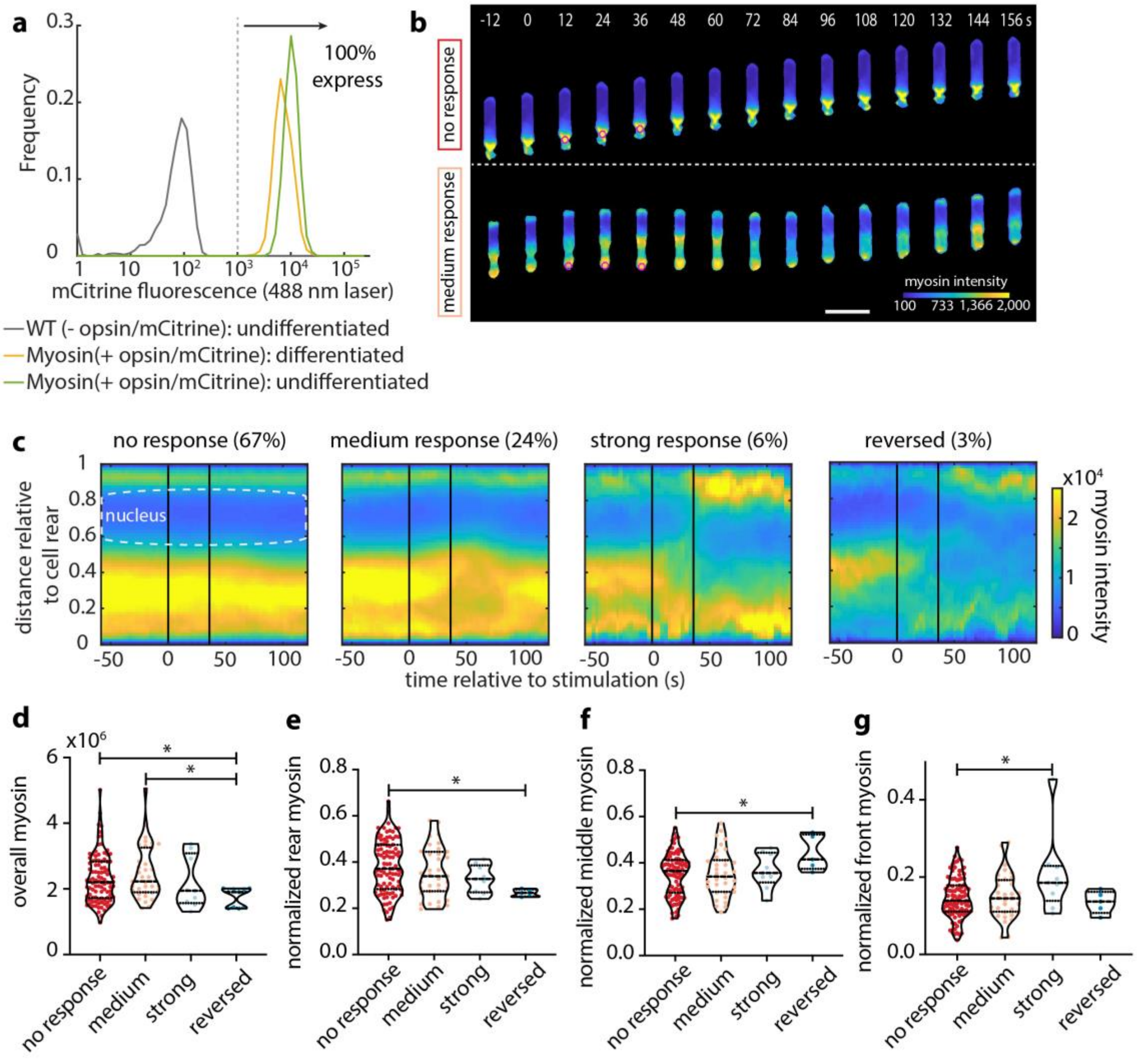

Supplementary Figure 5: Myosin quantification supports the idea that cellular responses are resulting from pre-existing variation.

(a) Flow cytometry measurement for mCitrine fluorescence of *n*=9990 differentiated (red) and *n*=10243 undifferentiated (purple) HL60 cells expressing parapinopsin (tagged with mCitrine), Myl9 and a cytosolic tag. Wild type cells, *n*=9904, not expressing the opsin used as control (grey). **(b)** Live-cell imaging snapshots of stimulation experiments with cells expressing parapinopsin and a myosin light chain sensor/cytosolic tag showing no response (upper panel) and a medium response (lower panel). Cells migrated unperturbed for 60 s prior to starting the transient 12-pulse stimulation at their cell rear (magenta circles). Myosin intensity pseudo colored to facilitate visualization. Images were captured every 3 s and subsampled for illustration purposes. Scale bar: 25 µm. **(c)** Average kymograph representation of myosin intensity as a function of time (*x*-axis) and vertical position relative to the cell rear (*y*-axis) of *n*=147 cells from 8 independent experiments stratified as non-responding, medium responding, strong responding and reversing cells. Vertical black lines indicate the start and end of the pulsated stimulation. **(d-g)** Violin plots of mean overall myosin **(d)**, mean normalized rear myosin **(e)**, mean normalized middle myosin **(f)**, and mean normalized front myosin **(g)** of *n*=147 cells, averaging over 15 s prior to initiating 12-pulse stimulation; *p*-values of two-sided Wilcoxon rank sum test (*: $p<0.05$, pairs not shown have $p>0.05$). Source data are provided as a Source Data file, including exact *p*-values.

Supplementary Figure 6

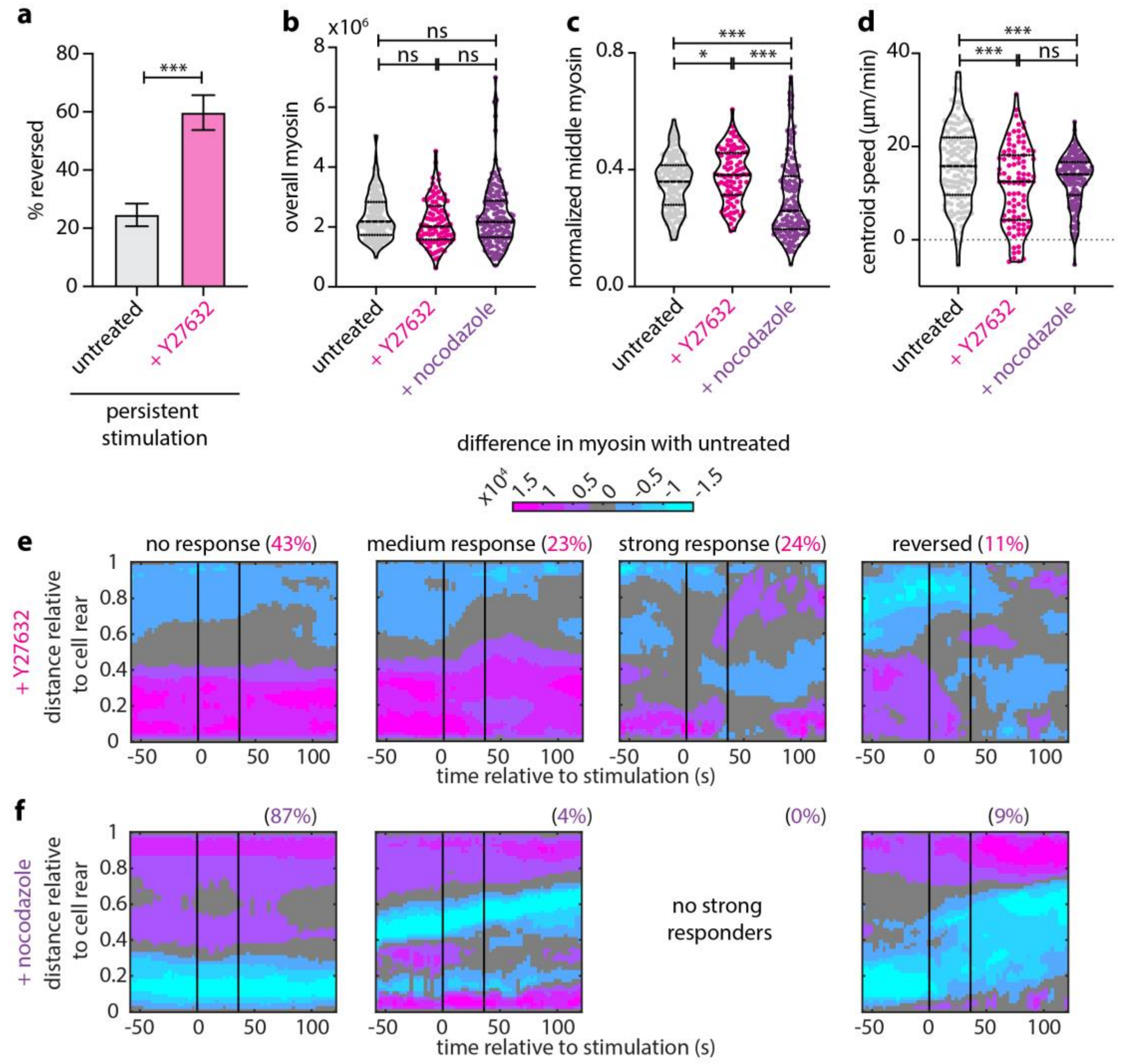

Supplementary Figure 6: Intracellular myosin localization is altered by perturbations affecting phosphorylation of myosin regulatory light chain.

(a) Bar plot of the percentage of myosin-expressing cells that reversed in response to persistent stimulation for n=122 untreated cells and n=67 Y27632-treated cells (from 5 and 4 independent experiments, respectively). Error bars represent confidence intervals assuming a binomial distribution around the cumulative mean of each group. Two-sided Fisher exact test revealed a significant difference between the two conditions (***: p<0.001). **(b-d)** Violin plots of mean overall myosin **(b)**, mean normalized cell middle myosin **(c)**, and mean centroid speed **(d)** for n=147 untreated cells, for n=93 Y27632-treated, and n=137 nocodazole-treated cells (from 8, 10 and 8 independent experiments, respectively), averaging over 15 s prior to initiating the 12-pulse stimulation; p-values of two-sided Wilcoxon rank sum test (*: p<0.05, ***: p<0.001, ns: p>0.05). **(e-f)** Average kymograph representation of the difference in myosin intensity as a function of time (x-axis) and vertical position relative to the cell rear (y-axis) between n=147 untreated cells and n=93 Y27832-treated **(e)**, and n=137 nocodazole-treated cells **(d)**, stratified as no responders, medium responders, strong responders and reversers (left to right) for 12-pulse stimulation assays. Vertical black lines indicate the start and end of the pulsated stimulation. Source data are provided as a Source Data file, including exact p-values.
