# Supplementary material for: Directional reorientation of migrating neutrophils is limited by suppression of receptor input signaling at the cell rear through myosin II activity
Source: Nat Commun. 2021 Nov 16;12:6619. doi: 10.1038/s41467-021-26622-z (PMC8595366; doi:10.1038/s41467-021-26622-z)
Supplement: Supplementary file 2 — Description of Additional Supplementary Files [file 41467_2021_26622_MOESM2_ESM.pdf]

Description of additional supplementary files

Title: Supplementary Data 1

Primers used in this work.

Title: Supplementary Movie 1

Description: A cell reversing its direction of motion under persistent optogenetic stimulation at the cell rear. A successful reversal of an HL60 cell expressing parapinopsin and the Cdc42 FRET sensor. The cell migrated unperturbed for 60 s before administering persistent optogenetic stimulation at its rear (magenta circle). On the left we show the registered sensors (grey scale) and on the right the computed Cdc42 activity. Images were captured every 3 s. Scale bar: 25 µm.

Title: Supplementary Movie 2

Description: A cell responding at the level of Cdc42 when stimulated at its center. Representative center stimulation experiment on an HL60 cell expressing parapinopsin and the Cdc42 FRET sensor. The cell migrated unperturbed for 60 s before administering 5 pulses at their centroid (magenta circle). On the left we show the registered sensors (grey scale) and on the right the computed Cdc42 activity. Images were captured every 3 s. Scale bar: 25 µm.

Title: Supplementary Movie 3

Description: Transient stimulation at the cell rear results in four distinct cellular responses. Transient stimulation experiment on four different cells expressing parapinopsin and the Cdc42 FRET sensor showing no response, medium response, strong response and reversed (left to right). Cells migrated unperturbed for 60 s prior to starting a transient 12-pulse stimulation at their cell rear (magenta circle). On the left we show the registered sensors (grey scale) and on the right the computed Cdc42 activity. Images were captured every 3 s. Scale bar: 25 µm.

Title: Supplementary Movie 4

Description: Myosin sub-behaviors under transient stimulation at the cell rear. Transient stimulation experiment on four different cells expressing parapinopsin and a myosin light chain sensor/cytosolic tag showing no response, medium response, strong response and reversed (left to right). Cells migrated unperturbed for 60 s prior to starting a transient 12-pulse stimulation at their cell rear (magenta circle). Raw myosin intensity on the left and pseudo colored on the right to facilitate visualization. Images were captured every 3 s. Scale bar: 25 µm.
