# Supplementary material for: Directional reorientation of migrating neutrophils is limited by suppression of receptor input signaling at the cell rear through myosin II activity
Source: Nat Commun. 2021 Nov 16;12:6619. doi: 10.1038/s41467-021-26622-z (PMC8595366; doi:10.1038/s41467-021-26622-z)
Supplement: Supplementary file 8 — Reporting Summary [file 41467_2021_26622_MOESM8_ESM.pdf]

nature portfolio

Corresponding author(s): S.R.C,J.A.T

Last updated by author(s): Sept 16,2021

Reporting Summary

Nature Portfolio wishes to improve the reproducibility of the work that we publish. This form provides structure for consistency and transparency in reporting. For further information on Nature Portfolio policies, see our Editorial Policies and the Editorial Policy Checklist.

Statistics

For all statistical analyses, confirm that the following items are present in the figure legend, table legend, main text, or Methods section.

n/a	Confirmed	
☐	☒	The exact sample size (*n*) for each experimental group/condition, given as a discrete number and unit of measurement
☐	☒	A statement on whether measurements were taken from distinct samples or whether the same sample was measured repeatedly
☐	☒	The statistical test(s) used AND whether they are one- or two-sided<br>*Only common tests should be described solely by name; describe more complex techniques in the Methods section.*
☒	☐	A description of all covariates tested
☐	☒	A description of any assumptions or corrections, such as tests of normality and adjustment for multiple comparisons
☐	☒	A full description of the statistical parameters including central tendency (e.g. means) or other basic estimates (e.g. regression coefficient) AND variation (e.g. standard deviation) or associated estimates of uncertainty (e.g. confidence intervals)
☐	☒	For null hypothesis testing, the test statistic (e.g. *F*, *t*, *r*) with confidence intervals, effect sizes, degrees of freedom and *P* value noted<br>*Give P values as exact values whenever suitable.*
☒	☐	For Bayesian analysis, information on the choice of priors and Markov chain Monte Carlo settings
☒	☐	For hierarchical and complex designs, identification of the appropriate level for tests and full reporting of outcomes
☐	☒	Estimates of effect sizes (e.g. Cohen's *d*, Pearson's *r*), indicating how they were calculated

Our web collection on statistics for biologists contains articles on many of the points above.

Software and code

Policy information about availability of computer code

Data collection	Micromanager (version 1.4.23) paired with custom MATLAB code (version R2015a) were used to automate experiments. The procedures executed by the code are described in the Methods, and the code is available upon reasonable request to the corresponding authors. Flow cytometry measurements were performed using a Becton-Dickinson (BD) FACSCanto II Flow Cytometer and data were collected using BD FACSdiva (version 6.1.3). Photolithography masks for the microfluidic devices were designed using AutoCAD (Autodesk).
Data analysis	Custom MATLAB code (version R2018a) was used for data analysis and is available from the corresponding authors upon reasonable request. Fisher exact test for contingent matrices larger than 2 by 2 was performed using R (RStudio version 0.99.893). Plotting was performed using MATLAB (R2018a) and GraphPad Prism 8.

For manuscripts utilizing custom algorithms or software that are central to the research but not yet described in published literature, software must be made available to editors and reviewers. We strongly encourage code deposition in a community repository (e.g. GitHub). See the Nature Portfolio guidelines for submitting code & software for further information.

Data

Policy information about availability of data

All manuscripts must include a data availability statement. This statement should provide the following information, where applicable:

- Accession codes, unique identifiers, or web links for publicly available datasets
- A description of any restrictions on data availability
- For clinical datasets or third party data, please ensure that the statement adheres to our policy

All processed data generated in this study are provided in the Supplementary Information and Source Data files. Due to the large size of the full dataset, raw images are not included but are available from the corresponding authors upon reasonable request. Source data are provided with this paper.

Field-specific reporting

Please select the one below that is the best fit for your research. If you are not sure, read the appropriate sections before making your selection.

☒ Life sciences ☐ Behavioural & social sciences ☐ Ecological, evolutionary & environmental sciences

For a reference copy of the document with all sections, see nature.com/documents/nr-reporting-summary-flat.pdf

Life sciences study design

All studies must disclose on these points even when the disclosure is negative.

Sample size	No sample size calculations were performed. Sample sizes were chosen based on practical limitations, we aimed for large numbers, especially for central experiments, to be sure that effects would be well resolved.
Data exclusions	Cells for which image segmentation and/or registration failed were removed.
Replication	All experiments were performed at least 3 independent times. In figure legends we denote the number of independent experiments performed in each case. We performed experiments multiple independent days and multiple times within the same day and the trends were always consistent. Any variability was consistent with Poisson counting statistics (see Methods).
Randomization	The choice for the microfluidic chip for each experiment was random, selection of individual cells could not be randomized because they had to be manually selected based on entry inside the channel but we carefully tested covariates such as distance migrated inside the channel to make sure that these were not sources of bias. Cells for which treatment would be applied were also selected randomly. Conditions to be compared were conducted back to back to minimize any potential bias.
Blinding	Blinding was not relevant for flow cytometry experiments, as the measurements are automated by the machine, the only manual step was gating for viable cells. An example of the gating strategy is included in Supplementary Information. All other experiments were imaging-based. For all of these experiments, imaging protocols were automated after cell selection. Cell selection had to be done manually so as to image cells that were fully inside the microfluidic channel.

Reporting for specific materials, systems and methods

We require information from authors about some types of materials, experimental systems and methods used in many studies. Here, indicate whether each material, system or method listed is relevant to your study. If you are not sure if a list item applies to your research, read the appropriate section before selecting a response.

Materials & experimental systems

n/a	Involved in the study	
☒	☐	Antibodies
☐	☒	Eukaryotic cell lines
☒	☐	Palaeontology and archaeology
☒	☐	Animals and other organisms
☒	☐	Human research participants
☒	☐	Clinical data
☒	☐	Dual use research of concern

Methods

n/a	Involved in the study	
☒	☐	ChIP-seq
☐	☒	Flow cytometry
☒	☐	MRI-based neuroimaging

Eukaryotic cell lines

Policy information about cell lines

Cell line source(s)	HL-60 cells were obtained from Orion Weiner's lab UCSF, who got them from Henry Bourne's lab.
Authentication	HL60 cells have highly characteristic behaviors, including rapid speed migration, elongated morphology, and responsiveness to formyl peptides. Cell lines were confirmed based on behavior.
Mycoplasma contamination	All lines tested negative for mycoplasma contamination.
Commonly misidentified lines (See ICLAC register)	There are no commonly misidentified cell lines used in the study.

Flow Cytometry

Plots

Confirm that:

☒ The axis labels state the marker and fluorochrome used (e.g. CD4-FITC).

☒ The axis scales are clearly visible. Include numbers along axes only for bottom left plot of group (a 'group' is an analysis of identical markers).

☒ All plots are contour plots with outliers or pseudocolor plots.

☒ A numerical value for number of cells or percentage (with statistics) is provided.

Methodology

Sample preparation	Cell lines were analyzed in FACs buffer (Phosphate buffered saline (PBS) + 5% FBS + 0.01% sodium azide).
Instrument	Becton-Dickinson (BD) FACSCanto II Flow Cytometer
Software	Data were collected using BD FACSDiva software (version 6.1.3) and analyzed using custom scripts in MATLAB (R2018a).
Cell population abundance	We only analyzed one cell population for each sample.
Gating strategy	Cells were gated using a polygonal FSC vs SSC gate to gate for viable cells. The gate was drawn manually to best separate the distinct two populations. A threshold for “positive” presence of mCitrine was manually set at 10^3 to separate the two peaks in a clearly bimodal distribution across samples. Supplementary Figure 1a exemplifies the gating strategy.

☒ Tick this box to confirm that a figure exemplifying the gating strategy is provided in the Supplementary Information.

March 2021
